# Racial and Ethnic Representativeness of California Medical Schools, 2021

**DOI:** 10.1001/jamanetworkopen.2022.51192

**Published:** 2023-01-13

**Authors:** Megan H. Yee, Richard L. Kravitz

**Affiliations:** 1 Elk Grove, California; 2 Department of Internal Medicine, UC Davis School of Medicine, Sacramento, California

## Abstract

This cross-sectional study evaluates representation of racial and ethnic groups underrepresented in medicine among students attending California medical schools based on whether the representation standard is calculated at the local, state, or national level.

## Introduction

Increasing the share of medical school graduates belonging to racial and ethnic groups underrepresented in medicine (URM) has been a longstanding national priority. In addition to rectifying historical discrimination in medical student admissions, arguments for diversifying the physician workforce include evidence that students from minority racial and ethnic groups are more likely to practice in underserved areas^1^; that such students may provide more culturally sensitive and higher quality care to patients from backgrounds similar to their own^2^; and that non-URM students benefit from learning alongside diverse colleagues.^3^ Thus, an emerging consensus is that graduates should represent the communities they serve.

In 2004, the Association of American Medical Colleges (AAMC) defined URM in medicine to mean “underrepresented in the medical profession relative to…the general population.”^4^ However, allowing that each medical school class should reflect the general population or community raises the question of which population or community? *US News and World Report* assesses medical school diversity in part by comparing a school’s URM proportion to the corresponding proportion of the state population (for public schools) or the US national population (for private schools). Others have called for parity with local or regional demographics.

Using 3 different population standards, we examined California’s allopathic medical schools for racial and ethnic representativeness. We hypothesized that the extent to which a medical school achieves its diversity goals depends on whether the population standard is local, state, or national.

## Methods

This study was judged exempt by the UC Davis institutional review board because it was not considered human participants research. It followed the Strengthening the Reporting of Observational Studies in Epidemiology (STROBE) reporting guideline for cross-sectional studies. The proportion of URM students enrolled at California’s 11 medical schools was obtained from the AAMC’s 2021 Enrollment Database, which uses matriculant self-report of race and ethnicity.^5^ The state’s newest school, California University of Science and Medicine, lacked a full complement of matriculants in 2021 and was therefore not included (Table).

**Table.** Representativeness Quotients and Relative Rankings for California Medical Schools Using Local (Metropolitan Statistical Area), State, and National Criteria for Students Underrepresented in Medicine^a^

School	URM %	Category	MSA RQ^a^	Rank	State RQ	Rank	United States RQ	Rank
UC Davis	28.9	Public	95.7	1	63.5	2	89.5	2
UCSF	22.3	Public	72.9	2	48.9	5	68.9	5
Stanford	16.3	Private	56.5	3	35.9	8	50.6	8
UC Riverside	31.6	Public	55.3	4	69.5	1	97.9	1
UCLA	26.5	Public	51.8	5	58.2	3	82.0	3
Kaiser-Permanente	24.0	Private	46.9	6	52.7	4	74.3	4
UC San Diego	14.7	Public	37.5	7	32.3	9	45.4	10
USC	19.0	Private	37.2	8	41.8	6	58.9	6
UC Irvine	17.9	Public	35.1	9	39.4	7	55.5	7
Loma Linda	15.5	Private	26.2	10	25.8	10	48.1	9
California Northstate	2.7	Private	8.9	11	5.9	11	8.3	11
Median			46.9		41.8		58.9	

Abbreviations: MSA, Metropolitan Statistical Area; RQ, representativeness quotient; UC, University of California; URM, underrepresented in medicine (students from minority racial and ethnic groups).

^a^ Spearman correlations between ranks for local-state, state-national, and local-national comparisons were 0.71 (*P* = .02), 0.99 (*P* < .001), and 0.68 (*P* = .02), respectively.

The URM proportion in the Metropolitan Statistical Area (MSA) of each medical school, in California as a whole, and in the United States were obtained from the 2020 US Census.^6^ URMs were defined as African American, Hispanic, Native American or Alaskan Native, and Native Hawaiian or Pacific Islander students. A representativeness quotient (RQ) was calculated by dividing the school’s percentage of URMs by the corresponding population percentage and multiplying by 100. RQs were analyzed by categories of greater than 80%, 50% to 79.9%, and less than 50%. Spearman correlation coefficients were calculated to compare RQ rankings among schools using local, state, and national criteria; 2-sided *P* < .05 were considered statistically significant. All statistical analyses were performed using Stata 15.1 (StataCorp) from June to November 2022.

## Results

The median (range) representativeness quotient (RQ) for URMs across the 11 schools was 46.9 (8.9-95.7) for local (MSA) population criteria, 41.8 (5.9-69.5) for state, and 58.9 (8.3-97.9) for national. Whereas no school achieved an RQ of at least 80 using state-level demographics as the criterion, 3 schools met this standard using national data (Table). For some medical schools, using different criteria shifted assessed performance. For example, Stanford University School of Medicine dropped 5 spots in the rankings when assessed using state or national rather than local data. In contrast, UC Riverside, UCLA, Kaiser-Permanente, and UC San Diego moved up by at least 2 places when state or national data were used in lieu of local data.

In data disaggregated by race and ethnicity, median (range) RQs across the 11 schools for African American students were 131.0 (11.0-340.6) (local), 155.7 (13.7-261.2) (state), and 65.6 (5.7-109.4) (national), indicating point-in-time high proportionality at the local and state levels and underrepresentation at the national level. The corresponding median (range) RQs for Hispanic or Latino were 29.0 (8.8-89.8), 29.3 (5.0-56.3), and 62.8 (15.3-120.6), respectively. Spearman rank order correlations for school-level RQ scores ranged from excellent (0.99 for URM representation using a state vs national reference standard) to extremely poor (0.03 for Hispanic representation using a state reference standard vs African American representation using a local standard).

## Discussion

In this cross-sectional study, an institution’s putative success at representing the communities they serve varied depending on: (1) use of local, state or national population data; and (2) focus on URMs in the aggregate vs specific racial and ethnic groups. Because California is demographically more diverse than most other states (a result of large Hispanic and Asian populations), URM RQs using national denominators tended to reflect more favorably on California medical schools’ recruitment efforts than URM RQs using California-specific denominators. However, because the percentage of African American students in California is about half the national average, the reverse was true for RQs restricted to African American.

More broadly, discussion is needed about which communities medical schools serve. *The US News* approach (ie, state standards for public schools, national standards for private schools) has been accepted without much fanfare but neglects the geographic concentration of medical schools in urban areas and the many advantages accorded private schools, including federal and state tax exemptions and other public subsidies.

Although this study was limited by analyzing a single state, we hope it spurs additional research and debate about the meaning of diversity, the aims of parity, and the use of metrics to measure success. In addition, the dissemination of these results may help inform the enrollment choices of prospective medical students.

## Notes

Supplement.
Data Sharing Statement
