# Supplementary material for: Racial and Ethnic Representativeness of California Medical Schools, 2021
Source: JAMA Netw Open. 2023 Jan 13;6(1):e2251192. doi: 10.1001/jamanetworkopen.2022.51192 (PMC9856646; doi:10.1001/jamanetworkopen.2022.51192)
Supplement: Supplement. — Data Sharing Statement [file jamanetwopen-e2251192-s001.pdf]

Data Sharing Statement

Yee. Racial and Ethnic Representativeness of California Medical Schools, 2021. *JAMA Netw Open*. Published January 13, 2023. doi:10.1001/jamanetworkopen.2022.51192

Data
Data available: No

Additional Information
Explanation for why data not available: All data are publicly available to anyone through the AAMC and the US census.
